# Fusion transcripts are present in early progenitor cells in *KMT2A*-rearranged B-ALL

**DOI:** 10.1038/s41375-024-02164-3

**Published:** 2024-02-02

**Authors:** Ricky Tirtakusuma, Paul Milne, Helen J. Blair, Yuzhe Shi, Simon Bomken, Olaf Heidenreich

**Affiliations:** 1 Wolfson Childhood Cancer Research Centre, Translation and Clinical Research Institute, Newcastle University Centre for Cancer, Newcastle Upon Tyne, UK; 2 Haematopoiesis and Immunogenomics Laboratory, Translational and Clinical Research Institute, Newcastle University, Newcastle upon Tyne, England; 3 Center for Cell Engineering and Immunology Program, Sloan Kettering Institute, Memorial Sloan Kettering Cancer Center, New York, NY USA; 4 Princess Máxima Center for Pediatric Oncology, Utrecht, The Netherlands

**Keywords:** Acute lymphocytic leukaemia, Oncogenes

## To the Editor:

While childhood B cell acute lymphoblastic leukemia (ALL) has an excellent prognosis at diagnosis, outcomes for relapsing patients have been disappointingly low. This grim picture has recently improved with the advent of bispecific T cell engagers (BiTEs) and chimeric antigen receptor (CAR) T cells targeting CD19 with, for instance, CART cells achieving complete remission in over 80% of patients with B-ALL. Nevertheless, 30–50% of the patients still experience relapse within one year [1] with three quarters of relapses showing loss of CD19 surface expression [2]. In particular *KMT2A*-rearrangements, which independently predict poor outcome [3], are prone to treatment failures resulting from lineage-switched CD19-negative relapse [4]. Recently, we demonstrated that lineage switch can originate either in the ALL blast population or from an immature progenitor population and that *KMT2A*-rearranged infant ALL is characterized by an early lymphocyte precursor (ELP) signature that was not detectable in the lineage-switched myeloid relapse [5, 6]. These results complement similar findings from mouse models of t(4;11) ALL [7, 8] and *TCF3::ZNF384* BCP-ALL also pointing towards an early progenitor with lymphoid potential pre-VDJ recombination [9, 10]. These combined findings raise the question about the nature of the cell of origin of this type of B-ALL. Here we demonstrate the presence of *KMT2A*-rearrangements in progenitor cells harboring both lymphoid and myeloid potential and their capacity to initiate leukemia, potentially acting as the cellular origin of CD19-negative relapse.

**Fig. 1:**
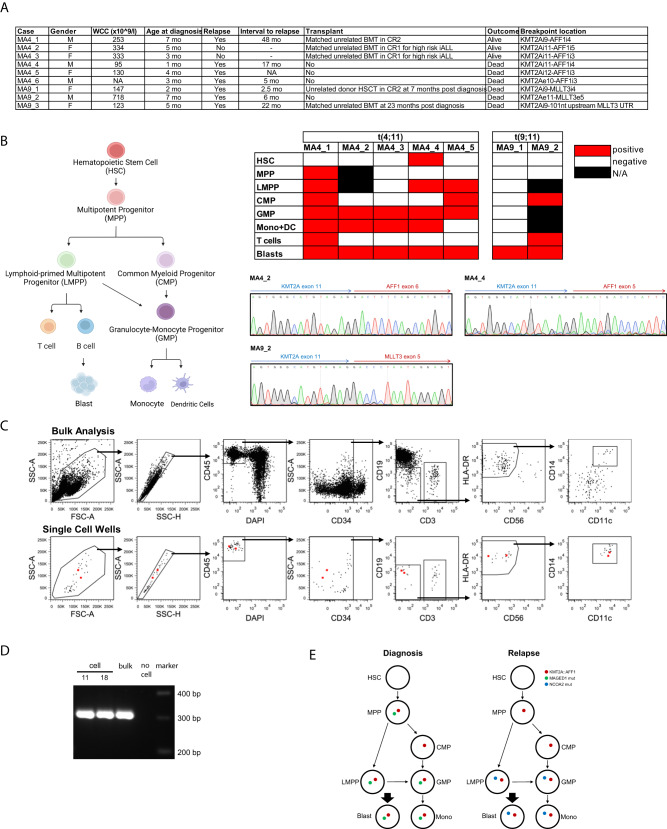
Identification of *KMT2A*-rearrangement in different hematopoietic subsets. **A** Clinical information patient cohort. **B** Summary of *KMT2A::AFF1* and *KMT2A::MLLT3* presence in different subsets of hematopoietic population. Representative patients MA4_2, MA4_4, and MA9_2 breakpoint sequences were shown by Sanger sequencing. **C** Single-cell sorting of patient MA4_1 for monocyte (HLA-DR+CD14+CD11c+) populations. The two *KMT2A::*AFF1-positive cells, cells 11 and 18, are highlighted in red throughout the sorting strategy (lower panel). **D** Amplification of the *KMT2A::AFF1* fusion in MA4_1 in cells 11, 18, and bulk sample shows the expected 299 bp band. **E** Summary *KMT2A::AFF1*, *MAGED1*, and *NCOA2* mutation assessment on sorted populations MA4_1 diagnosis and relapse. *MAGED1* is mutated gene only found at diagnosis. *NCOA2* is mutated gene only found at relapse.

We examined seven infants with ALL and *KMT2A*-rearrangements at diagnosis: five with *KMT2A::AFF1* and two with *KMT2A::MLLT3* fusions (Fig. 1A, B). The following hematopoietic stem and progenitor cell populations were isolated: HSCs (CD34+CD38−CD45RA−CD90+), multipotent progenitor cells (MPPs, CD34+CD38−CD45RA−CD90−), lymphoid-primed multipotent progenitor cells (LMPPs, CD34+CD38−CD45RA+), common myeloid progenitor cells (CMPs), granulocyte monocyte progenitor cells (GMPs), mature monocytes, and T cells. We evaluated the presence of fusion genes in these purified populations by PCR. In three cases, the *KMT2A::AFF1* fusion gene was found in LMPPs. In two cases, *KMT2A*::*AFF1*-positive cells were also present either in the MPP or HSC fraction. In two *KMT2A*::*MLLT3* samples we detected only fusion gene-positive CMP-like and blast populations (Fig. 1B). Notably, the two *KMT2A::AFF1*-positive patients MA4_2 and MA4_3, had undetectable levels of the fusion gene in the HSC-MPP-LMPP subsets and did not undergo relapse (Fig. 1A, B). However, a larger cohort is required to follow up if there was any correlation between fusion gene positivity in early progenitor populations and incidence of relapse. Moreover, it is important to note that a negative PCR result does not formally exclude the presence the corresponding fusion gene at levels below the detection limits of the assay.

In concordance with the translocation occurring in early progenitor populations, we identified the *KMT2A::AFF1* fusion gene also in CD34–CD19–CD3–HLA-DR+ monocytes/dendritic cells in 4 of 5 cases, providing further evidence of an early *KMT2A-r* progenitor with bilineage, i.e., lymphoid and myeloid, potential (Fig. 1B). To exclude the possibility of sorting impurities, these results were confirmed by single-cell PCR of sample MA4_1. Monocytes were sorted into 96-well plates, followed by whole-genome amplification and PCR amplification of *KMT2A::AFF1*. The causative translocation was identified in 2 of 22 monocytes analyzed (Fig. 1C, D). These results imply an early *KMT2A::AFF1* progenitor-like cell with both lymphoid and myeloid potential that might serve as a source for lineage switch.

Indeed, patient MA4_1’s disease relapsed four years after diagnosis with an AML harboring the same *KMT2A::AFF1* breakpoint [5, 6]. We performed bulk whole-exome sequencing and identified, in addition to the shared *KMT2A::AFF1* fusion gene, mutations that were exclusively present at diagnosis (*MAGED1*) or at relapse (*NCOA2*). Assessment of each of these mutations in sorted cell populations detected *KMT2A::AFF1* in the MPP populations of both presentation and relapse (Fig. 1E). Mutated *MAGED1* was present in all diagnostic progenitor populations except HSCs and CMP-like cells. In contrast, mutated *NCOA2* was found in LMPP and GMP-like populations, but not in more immature cell populations (Fig. 1E). These findings identify a *KMT2A::*AFF1-positive cell as the cell of origin for both diagnostic ALL and relapse AML and show that secondary mutations were acquired at later stages. These data are also supported by our previous observation that immunoglobulin rearrangements were detected only at diagnosis but not at relapse of patient MA4_1 [6]. Therefore, these data suggest that the cell of origin had not initiated VDJ rearrangement, suggesting an MPP-like or even more immature phenotype.

**Fig. 2:**
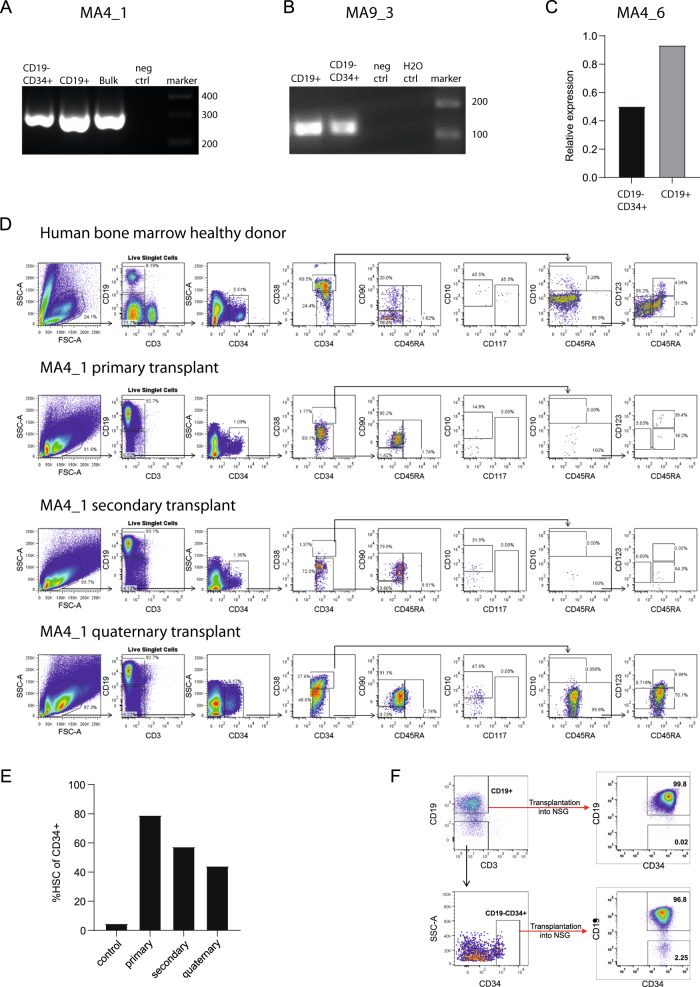
CD19^−^ CD34^+^ population can reconstitute CD19^+^ and CD19^−^ blasts. PCR identification of **A** *KMT2A::AFF1* and **B** *KMT2A::MLLT3* fusions within sorted human CD19–CD34+ and CD19+ on PDX MA4_1 and MA9_3, respectively. 293T cells provided a fusion gene negative control. **C** qRT-PCR analysis of *KMT2A::AFF1* expression in CD34+CD19- and CD19+ cells from PDX MA4_6. **D** Flow cytometric analysis of bone marrow-derived hematopoietic precursors from normal human bone marrow control, and across four generations of NSG mouse xenografts. The CD34+CD38-CD45RA-CD90 + HSC population is expanded and maintained across four generations. **E** Proportion CD34^+^CD38^−^CD45RA^−^CD90^+^ HSC gate of CD19− CD34+ cells patient MA4_1 across sequential transplantation. **F** Sorting of CD34+CD19− and CD19+ populations from patient MA4_1, left panel. Both populations were transplanted into NSG and evaluated following the engraftment, right panel.

To further characterize and functionally investigate the *KMT2A*-rearranged precursor cells, we generated patient-derived xenograft (PDX) models by transplanting the unsorted diagnostic samples into NOD-scid/IL2Rγ–/– (NSG) mice. Initially we selected two *KMT2A::AFF1* (MA4_1 and MA4_6) samples and one *KMT2A::MLLT3* (MA9_3) sample. Following engraftment, haematopoietic cells were collected and analyzed by flow cytometry. We observed a CD19+ blast population and, in addition, a more immature CD34+CD19– population. These populations were sorted, and the fusion gene expression was assessed by qPCR. Fusion gene transcripts were observed in CD34+CD19– cells in all three samples (Fig. 2A–C), albeit ~2-fold lower compared to the CD19+ compartment (Fig. 2C). Notably, serial transplantation across 4 generations of mice maintained this human HSC compartment confirming its self-renewal potential (Fig. 2D, E). Finally, we isolated CD19+ and CD34+CD19− populations and transplanted them into NSG mice. We observed that the CD34+CD19− population could reconstitute the disease by having both CD19+ and CD19– subsets (Fig. 2F), consistent with previous studies [8, 9].

This study confirms recent findings that the cell of origin in B-ALL is located at an early progenitor stage preceding the ELP stage and may be of pre-leukemic nature [5, 7, 11]. CD19-negative populations contain *KMT2A::AFF1* or *KMT2A::MLLT3* fusion genes and can progress towards malignancy, raising the question of the nature of factors co-operating with *KMT2A*-rearrangements to produce full transformation. Our data support the need to involve other targets, such as dual CD19/CD22, to prevent relapse caused by CD19-negative cells. The involvement of CD22 is of potential interest because its expression starts at the LMPP stage [11]. Alternatively, we propose targeting the *KMT2A* fusion gene, which is present in both CD19+ and CD19– populations at the transcript level or via fusion peptides presented by major histocompatibility complex and recognized by T cells [12–15]. These studies support the feasibility of discovering other fusion gene–reactive T cells, including reactivity for *KMT2A*-rearrangements.
